# Consumption Preferences of Pulses in the Diet of Polish People: Motives and Barriers to Replace Animal Protein with Vegetable Protein

**DOI:** 10.3390/nu13020454

**Published:** 2021-01-29

**Authors:** Magdalena Śmiglak-Krajewska, Julia Wojciechowska-Solis

**Affiliations:** 1 Department of Finance and Accounting, Faculty of Economics, University of Life Sciences, Wojska Polskiego 28, 60-637 Poznan, Poland; smiglak@up.poznan.pl; 2 Department of Agritourism and Rural Development, Faculty of Agrobioengineering, University of Life Sciences in Lublin, Akademicka 13, 20-950 Lublin, Poland

**Keywords:** pulses, consumption, nutrition intentions, attitudes, food culture, food choice, sustainable diets, healthy eating plate

## Abstract

Today, with easy access to information, people are aware of the health benefits of pulses and their significant role in sustainable consumption. Despite this, the consumption of pulses still remains low in many developed and developing countries. The authors of the study attempted to fill the gaps in knowledge about determinants that influence the consumption of pulses. The study was conducted to identify the motives and barriers which, according to a consumer, influence the level of consumption of pulses. An attempt was made to determine to what extent a consumer is influenced by culinary trends (using the multiple linear regression equation). The survey was conducted on a sample of 1067 Polish respondents. The sample was representative and selected to reflect the social demographic distribution of the respondents. In the research, descriptive statistics were used, as well as a *t*-test, linear regression statistics, and discriminant function analysis. Pulses are more often considered by women to be healthy and nutritious products as well as a good alternative to meat products. The price of pulses products is very important. Men also consider those qualities to be encouraging to consume pulses, but to a lesser extent than women. The lack of knowledge and skills to prepare tasty meals from pulses is one of the most important barriers which, both in the case of women and men, prevents consumers from eating pulses more often. Identified types of diets of the respondents in the research sample showed differences in the regularity of consumption of different selected pulse products. The obtained results indicated that Polish consumers are influenced by other cuisines of the world in their preferences. The most influential cuisines were the Middle Eastern and Mediterranean, with a variety of dishes that are prepared based on pulse products. It should be emphasized that to increase the consumption of pulses, it is necessary to continue educating society through various channels, e.g., culinary, health, information programs, or by inviting celebrities who enjoy the great trust of the society to take part in the advertisement.

## 1. Introduction

The essence of proper nutrition is to provide the body with all the necessary nutrients in appropriate quantities and proportions. One of the groups of plants that are valuable from a nutritional point of view are pulses. These plants are a rich source of plant proteins, minerals, fiber, biologically active substances, and vitamins [1,2]. In many regions of the world, pulses are a unique source of protein in the diet [3]. Very often they are an essential supplement to other sources of protein. Therefore, the dietary importance of pulses is expected to increase in the coming years to cover the protein requirement (and other nutrients) of the growing world population and the need to reduce the risks associated with the consumption of animal food, especially in developed countries [4].

Pulses are quite a diverse group. They are cultivated as a forecrop to fertilize the soil, raw material for the production of animal feed, and an edible product for humans which is a rich source of protein and other nutrients [5]. The group of pulses, according to FAO, include 12 species harvested for dry seed. The classification excludes fresh pulses and plants that are used mainly for the extraction of oil and for sowing purposes [6]. The most popular species in Poland, and most often consumed are: Dry beans, pea, broad bean, soybean, chickpeas, and lentils.

Vegetable protein has a different composition of amino acids than animal protein derived from milk and meat. The nutritional value of the protein contained in pulse products is only slightly lower than the nutritional quality of meat protein; therefore, according to the general recommendations regarding the rules of healthy eating, meat should be partially replaced with pulses [7]. By replacing even a small fraction of the calories consumed from animal protein per day with the same amount of calories from plant protein, the risk of premature death is reduced [8]. Pulses contain the biggest amount of protein among all cultivated plants, i.e., from 20 to 42%, depending on the species and growing conditions [9,10,11,12]. Moreover, the biological value of the protein is higher than that of cereal protein, due to which the seeds are a valuable food for people and rich feed for animals and they are used in various branches of the food industry. To improve the profile of amino acids and to obtain complete protein, pulses should be combined with cereal grains that will supplement the missing amino acids. Dry soya beans contain complete proteins with a composition similar to animal protein [13]. Proteins contained in the products of animal origin are characterized by high nutritional value, i.e., they are complete proteins. The content of protein in pork is 15–21%, in beef it is 15–21%, and in poultry it is 18–23%.

An important factor that influences the nutritional value of a protein is its digestibility. According to the protein digestibility corrected amino acid score (PDCAAS) method, the average value of the index for plant proteins is 0.91 for soybeans, 0.78 for chick-peas, 0.75 for black beans, and 0.67 for peas. On the other hand, the proteins in the products of animal origin are characterized by a slightly higher level of assimilability, e.g., beef 0.92; chicken 0.95 [14].

Pulses are also a rich source of dietary fiber. The daily adult intake of fiber, recommended by the World Health Organization (WHO), should be between 20–40 g [15,16]. The Institute of Medicine of the National Academy of Sciences 2005 [17] specifies the recommendations at 14 g per 1000 calories consumed [18,19,20,21,22]. The high content of carbohydrates, as well as dietary fiber, make pulses a product with a low glycemic index [23,24,25]. Pulses are also a valuable source of minerals and B group vitamins [26,27,28,29,30]. They support hormonal regulation and protect women from the development of hormone-related tumors such as breast cancer and endometrium [31,32,33,34].

Food-based dietary guidelines are national guidelines that translate the recommended levels of nutrients in relation to food products, taking into account the specific nutritional and health requirements of the population of the country, the availability of different food products, and historical and cultural food traditions. With the help of the FAO, approximately 100 countries have developed food-based dietary guidelines. The evaluation of those guidelines shows that around 87 percent of the countries recommend including pulses in the diet regularly [6]. According to the dietary recommendations of the United States Department of Agriculture, presented graphically as the “healthy eating plate”, which is a supplement to MyPlate, it is recommended that vegetables and fruits constitute half the plate, whole-grain products 1/4 of the plate, and the rest of the plate should contain products that are a source of protein [35,36,37,38]. Currently, there are many countries (e.g., United Kingdom, Germany, Portugal) that depart from the concept of the pyramid in favor of the plate. The reason for departing from the “healthy eating pyramid” was primarily the desire to simplify the recommendations and to present them in a form that everyone could understand. It turned out that the pyramid was often misinterpreted, e.g., the products that were placed at the top of the pyramid were often considered to be the most important and vice versa—those at the bottom (i.e., vegetables, fruit, cereal products) were considered to be the least important. 

According to the National Food and Nutrition Institute in Poland, in the case of adults, the consumption of plant proteins constitutes two-thirds of the total amount of protein consumed in daily ration, and in the case of children and adolescents, it is no less than half of the total amount of protein consumed. Therefore, one of the principles of healthy nutrition is to limit the amount of meat consumption and replace it with dry pulses and fish [39]. According to Turlejska and co-authors, the daily consumption of dry pulses, depending on age and gender, should be between 5–15 g/day [40].

Globally, the average level of consumption of pulses is about 21 g per person per day. There are significant differences in the levels of consumption between regions and countries, and within countries there are differences between socio-economic classes [6]. In recent years, the consumption of pulses in Polish households has been clearly declining—in the case of families with farms and it remains stable among pensioners; however, the active city dwellers are less and less likely to reach for those vegetables [7]. The area of legumes sown for seeds has remained in the world at a similar level for several years and ranged from 61 to 70 million ha. The area sown for pulses in Europe is only 3% of the world area. In recent years, the European Union has been aiming to increase the production of pulses. Poland ranks fourth, after Great Britain and Lithuania, among the producers of pulses in the European Union. The largest area among the EU countries sown with pules belongs to Spain, followed by Poland [41,42]. The area of edible pulses sown in Poland in 2018 decreased, compared to the previous year, by 5.3 thousand hectares, i.e., by 7.5%, and amounted to 66 thousand ha [43].

Despite the wide range of health and nutritional benefits resulting from regular consumption of pulses, they are still not very popular. It is worth emphasizing that some health-promoting nutritional systems, such as the Mediterranean diet (pulses should be included in the menu three times a week) or the DASH (Dietary Approaches to Stop Hypertension) diet, might not be strictly vegetarian but they are based, for the most part, on vegetable foods [44]. Nevertheless, in recent years there has been a clearly greater interest in the consumption of pulses and in plant-based diets, which is undoubtedly the result of growing nutrition awareness of consumers on the one hand, and on the other hand, wide access to the knowledge about proper diet (the internet, books, TV programs) [45]. Today, lentils are widely used in Arabic and Indian cuisine. Lentils can be made into flour which is used, e.g., in India, for bread-baking. The Germans serve it as a New Year’s Eve dish, believing that it will ensure the prosperity of their house. Chickpeas are an ingredient of traditional Indian dishes, as well as Pakistani and Arabic cuisine. In Poland, it has gained popularity only recently, along with the promotion of Arabic and Turkish cuisine. The most famous chickpea dish is falafel, often served as a vegan meat alternative [46].

The guidelines for increasing the consumption of pulses are justified in terms of nutrition but also by socio-economic and environmental reasons. Cultivation of those plants is many times cheaper than meat production, especially in terms of water and soil efficiency, and it contributes to the reduction of greenhouse gas emissions [47,48,49]. Pulses have a significant impact on the reduction of hunger and malnutrition, ensuring food security and improving human health and the environment. Therefore, increasing consumption of pulses is an important element of the transition towards a more balanced and healthy diet. The aim of the article was to provide information on the preferences of Polish consumers regarding pulse products to understand the motives and barriers of consumption of pulse products; to identify the best purchase channels for pulse products; and to determine the impact of fashionable food trends (world cuisines) on broadening nutritional horizons. These facts would confirm the willingness of Polish consumers to buy and consume pulses in a wider time horizon.

## 2. Materials and Methods 

### 2.1. Sample and Study Design

The research consisted of 3 parts: A part that concerned producers (farmers producing pulses), processing plants, and the final purchaser—the consumer of pulse products.

The first and second stages concerning the producers and the processors was performed by the University of Life Sciences in Poznań and financed by the Multiannual Programme of the Ministry of Agriculture and Rural Development, and was entitled: “Increasing the use of domestic vegetable protein for the production of high-quality animal products under conditions of sustainable development”. The stages were realized in 2016–2020.

The third stage of the research has been conducted from December 2019 to February 2020. The survey consisted of 18 questions. The questions pertained to consumer’s knowledge about pulses, the health benefits thereof, dietary preferences, reasons for paying attention to pulses, the frequency of consuming meals made of these products, as well as other aspects. This article presented the results of the part of the questionnaire which concerned the dietary preferences of the respondents and the possibility of buying pulses. In order to conduct the survey using the computer-assisted web interview (CAWI) method with the application of all presented criteria, the authors used a database purchased for the purposes of the implementation of earlier RKU/DS/2 projects within the framework on the Department of Agritourism and Rural Development of the University of Life Sciences in Lublin. In order to maintain the methodological assumptions, a detailed selection of respondents and contact for data collection were commissioned to a research agency.

In the survey conducted among consumers, the authors used a diagnostic survey together with an original questionnaire (due to the country in which the survey was conducted, the questionnaire was prepared in Polish). The questionnaire was composed of three sections containing data on demographic statistics of the survey participants, their knowledge regarding pulse products, and their identification and willingness to consume them (most frequently bought products). 

The original questionnaire mainly used Likert’s five-point scale, containing the following categories of answers: “I definitely do not agree”, “I do not agree”, “I neither agree nor disagree”, “I agree”, and “I definitely agree”. In addition, attitudes were measured with the use of a five-level rank scale with the application of the construction and validation procedure. The frequency with which a given product was consumed was measured with the use of a 0–5 scale, where 0 stands for “I do not eat it”, 1—“I eat it more rarely than once a month”, 2—“I eat it once or twice a month”, 3—“I eat it once or twice a week”, 4—“I eat it three or four times a week”, and 5—“I eat it every day”. The alpha Cronbach test was used to evaluate the reliability of the measurement scales. The authors adopted the value of 0.85, which refers to the correctness of scale reliability. For the determination of the sample size, the adopted level of confidence was 0.95, the estimated fraction size was 0.50, and the tolerable error was 0.03. The test sample was selected from the entire adult population of Poland, which amounted to 31,532,048 people [44]. The size of the sample amounted to 1067 respondents (1618 surveys were sent to randomly selected respondents—only 1067 were completed properly; the other questionnaires were rejected), who were selected on the basis of their place of residence (country, towns of less than 30,000 residents and above 30,000 residents), age (up to 25, 26, up to 40, 41, and up to 55, 56 and more), as well as sex. The respondents resided in 6 regions of Poland. The number of respondents constituted representative samples of the following regions: The central region—220, the southern region—222, the eastern region—188, the north-western region—109, the north-eastern region—164, and the northern region—164. Women constituted 52.3% of the respondents and men—47.7%. The respondents were also asked to specify the dietary regimen which they followed or lack of one (Table 1).

### 2.2. Statistical Analyses

The Statistica 13.1 PL program (StatSoft Inc., Tulsa, OK, USA) was used to perform statistical analyses. In order to decide which variables distinguished two naturally emerging groups, the authors applied the discriminant function analysis which enabled them to analyze differences between groups of objects on the basis of a set of selected independent variables (predictors). In addition, the analysis was used in correlation studies, i.e., when causal relationships between variables were not well-known. A classification function in the form of a calculation of a coefficient defined for each group of variables was used in the study. In data analysis, the authors used descriptive statistics, a *t*-test, and linear regression statistics, in order to find the equation that best predicted the dependent variable as a linear function of independent variables. The following formula for multiple linear regression was used in this work:

Y = b0 + β1 ∗ x1 + β2 ∗ x2 +… + βk ∗ xk + ε

b0—constant; 

βi—model parameter (regression factors) describing the impact of the i-th variable;

β1, …, βk—partial regression factors;

x1, …, xk—tested variable;

ε—random component (Se).

Before commencing the analyses, the authors studied multidimensional normality, verifying the normality of each variable’s distribution. It was assumed that variable variance matrices were homogeneous in groups. Standard variation was not taken into account due to the large number of respondents in particular groups. Differences in averages with randomness probability of less than <0.05 were deemed to be statistically significant.

The Ehrenberg [50] duplication method was adapted to the needs of a given survey and used to study the purchase channels of pulse products. It consisted in showing the percentage of consumers who while shopping through channel A also shopped through channel B.

The following research questions were asked for verification:

RQ1: What pulse product, among the ones listed in the questionnaire, is the most popular one among consumers?

RQ2: Is the frequency with which meals containing pulse products are consumed satisfactory?

RQ3: Do men, like women, pay attention to health qualities when they buy and consume pulse products?

RQ4: Do the consumption barriers for pulse products mean the same for both sexes?

RQ5: Are consumers willing to buy pulse products using various purchase channels? 

RQ6: Are products that are more processed (level of readiness for consumption) more desired by consumers?

RQ7: Do the popular cuisines of the world have an impact on consumer’s behaviors in the market of pulse products? What cuisine has a greater impact on the diet of Poles compared to other currently popular global trends?

## 3. Results

### 3.1. Frequency of Pulse Products Consumption

**Figure 1:**
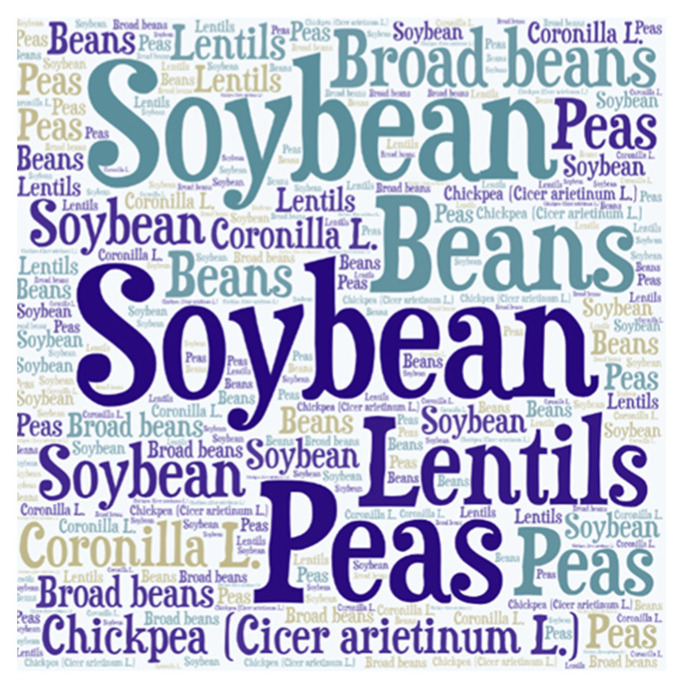
Word cloud—pulses preferences in the shopping cart one week before the survey. Note: A word cloud based on associations with legumes. The associations are based on the first 3 answers to the question: “What kind of pulses do I buy for preparing meals”. The more duplications there were, the bigger the word and the part is presented in the cloud. Source: Authors’ own results.

The respondents were asked to name at least 3 pulses that could be found in their shopping basket (at least one week before their participation in the survey). The cloud of words (font size and number of repetitions in the background depended on the number of times a given product was mentioned) showed that consumers most frequently selected products containing soybeans (e.g., soya milk, grains, tofu cottage cheese, half-products, e.g., soya chops), followed by products containing peas—32% (various frozen foods and canned vegetables) and beans—28% (ready meals in jars, e.g., Breton beans, frozen and dry products). The least often selected product were chickpeas, i.e., 10% (hummus), and broad beans, due to the seasonality of this plant in Polish cuisine (Figure 1). 

Analyzing the results, we can conclude that the average consumption of pulses in the Polish population is relatively low (Table 2). Foods made of pulses are consumed rarely, i.e., less than once a week (2.14). Among the products listed in the questionnaire, peas and beans were the ones to be mentioned most often by the respondents. People belonging to profile A (not following any dietary regimen) consumed high-protein plant products least often—they constituted 67.29% of the sample. Among persons who had to follow a specific dietary regimen for health reasons or reasons based on the ideology of the healthy lifestyle trend, the average consumption of pulses was from 1–2 a week to 3–4 times a week (from 2.55 to 3.60 on average). Soya and lentils are most popular among the consumers of profiles B and D, i.e., those following the “limit animal product” diet and “a special diet related to medical conditions”. Broad beans are the least popular plant among most consumers, except for people following specific dietary regimens. Despite the variety of dietary regimens followed by consumers, differences in the frequency with which meals containing pulses are consumed were statistically significant.

### 3.2. Motivation and Barriers for Purchasing and Consuming Pulses

Considering the sex of the respondents, the authors of the study obtained information regarding their motivation impacting their decisions on the consumption of pulses. The proposed model of discriminant analysis included five variables. Table 3 contained a detailed presentation of statistically significant discriminant differences in the studied groups in terms of motivation impacting decisions regarding consumption. The significance of the variables examined in the hierarchy—from the highest to the lowest analysis value—was as follows: “The products are healthy and nutritious” (F = 12.845; *p* = 0.001), “Pulse products are attractive in terms of their prices” (F = 8.809; *p* = 0.021); “They are meat substitutes and contain a lot of protein” (F = 7.281; *p* = 0.045); “They regulate blood sugar levels” (F = 5.410; *p* = 0.089); “They are part of my standard diet” (F = 3.357; *p* = 0.214). Nonetheless, the last two variables included in the model, which determined the reasons for consumption, showed no statistically significant differences. The classification function reached the highest value in the case of the “They are meat substitutes and contain a lot of protein” variable. This variable was most important for both men (2.091) and women (2.876). This may be because it is mostly women who are responsible for preparing meals at home, including making family meals more varied. With cooking shows being more popular than ever, as well as healthy eating trends and sustainable development social campaigns encouraging people to replace animal protein with plant protein being equally prevalent, pulse products successfully serve as a meat substitute for an increasing number of people. It is cooking shows that teach one how to prepare attractive dishes and use inexpensive ingredients to diversify one’s diet that have popularized pulse products in the first place. Regarding the “Pulse products are healthy and nutritious” variable, women (2.693) were more likely to pay attention to this aspect than men (2.133). A woman being responsible for the well-being of all household members is a widely-accepted Polish custom. Women (2.416) were also more interested in the price attractiveness of pulses than men (2.085); this may be because women are more likely to do shopping while also planning what meals to prepare at the same time. The fact that pulse products regulate blood sugar levels was of little importance to the entire population studied. The results indicated that this variable was only relevant for people who followed special diets aimed at lowering their blood sugar levels (e.g., diabetics), with this property of pulses being more important for men (1.501) than for women (1.404); however, the results were very similar. Pulses being part of one’s diet was also more important for men using them (1.345) than for women (1.168).

The respondents were also asked to indicate what they believe to be the most important barriers to pulse product consumption (Table 4). The statistically significant variables that determined consumption barriers in discriminatory analysis—from the highest to the lowest—were as follows: “Product taste and structure” (F = 7.845; *p* = 0.001), “Lack of knowledge and skills to prepare meals”. (F = 5.809; *p* = 0.006), “Pulse products contain a lot of carbohydrates” (F = 5.281; *p* = 0.004), “No such dietary habits” (F = 3.460; *p* = 0.028), “They cause bowel movements” (F = 3.357; *p* = 0.044). The classification function reached its highest values in the case of the “Lack of knowledge and skills to prepare meals” variable. This answer was more often indicated by women (2.186) than men (2.005), as it is mostly women who are responsible for preparing meals at home. However, men were in favor of meals based on pulse products (1.319) because compared to women (1.693), they did not consider the peculiar taste and structure of pulse product-based meals to be too much of a barrier. The claim that pulses contain high levels of carbohydrates was a similar barrier for both women (1.016) and men (1.011) included in the test sample. While pulse products are experiencing their “renaissance”, most consumers underestimate their health benefits, and as such, their presence is limited in people’s daily diets. The lack of dietary habits of eating meals with pulses is a much greater barrier for men (1.201) than for women (0.934); men often lack the knowledge on meals including such products, while women are quicker to include new foods in their menus. Bowel movements caused by pulses were a greater obstacle for women (0.908) than for men (0.745).

### 3.3. Purchase Preferences Concerning Pulse Products

Data in Table 5, in the A–H columns (see column names in the note, Table 5), show the percentage of consumers in the given purchase channel row who also used the purchase channels listed in the columns. For example, 27.85% of consumers buying pulses through channel B (producer stores) also purchased them through channel A (directly from farmers). More than half of the consumers who prefer to buy directly from farmers also bought pulses through channel C (markets, bazaars). It should be noted that pulse product consumers are well-informed and utilize all purchase channels available. Globalization, infrastructure development, and technical security have made channel H (Internet) popular among more than 8% of Polish food consumers, who use it as an alternative to other shopping channels (see average duplication). Respondents who use “specialized ecological stores” most often use channel H as well. It should be mentioned that the popularity of the “internet” channel has significantly increased since the beginning of the coronavirus pandemic. The most popular choices in the research sample included the “Specialized Healthy Food Stores—I Love Vege”, as well as festivals organized by local producers (held between June and September in all regions of Poland). While traditional markets and bazaars offer locally-sourced high-protein products, less than 40% of respondents take advantage of what they offer.

In this study, the following products were selected to be purchased by the consumer (Table 6): Fresh vegetables, frozen products, dried seeds, canned vegetables and salad mixes, and semi-processed/processed food in jars (e.g., hummus, baked beans in tomato sauce, and mixed vegetables). Women purchase pulse products much more often than men. Although men prefer ready-to-cook food over other products, only the fresh and frozen vegetable options are not statistically significant if a sex-differentiating variable is applied. As shown in Table 6, the degree of the product’s preparation for consumption is vital for consumers as it enables them to save time when preparing meals.

To determine which of the world cuisines has the greatest impact on the pulse product market nutritional behaviors, the respondents were asked to evaluate the following statement: “My favorite pulse dishes from various world cuisines affect my willingness to consume pulse products” (answer suggestions were coded in a five-point scale). The data were analyzed using a linear regression procedure and a set of factors obtained through factor analysis for each sample group was used as predictors. By performing multiple regressions, the authors wanted to find and explain the relationship between independent variables and dependent variables.

The coefficient of determination R^2^ is 0.322, which meant that the model explained the relationships between the variables in 32.2%. Among the suggested variables that determined the impact of dietary trends in the form of popular world cuisines, the model included five variables, and they are presented in Table 7.

The combination of variables that are presented in the table showed the expected contribution of different cuisine trends (world cuisines) that use pulses in prepared meals, and all five suggested variables have a significant impact on the expected pattern of consumer nutrition. The values β were as follows: The largest coefficient indicating which independent variable had the greatest influence on the dependent variable was “Middle Eastern cuisine” (C), then “Mediterranean Cuisine” (D), “Mexican Cuisine” (A), and “Indian Cuisine” (B), and at the end “Other cuisines of the world” (E). The regression equation was as follows:

Y = 2.587 + 0.107A + 0.086B + 0.120C + 0.113D + 0.061E ± 1.315

On the basis of the presented equation, we could answer the research question about the kitchen, which had a greater impact on the diet of Poles compared to others. Middle Eastern and Mediterranean cuisine was more important for the presence of pulses in the diet.

Summarizing the results obtained, it can be stated that Polish consumers are positively attracted to purchase and consume legume seeds in a wider time horizon.

## 4. Discussion

The conducted research provided information on the attitudes, barriers, and motives of consumption of pulses among Polish consumers. The studies of Jallinoja et.al [51] draw attention to the problem of Europeans related to the frequency of consumption of proteins of plant origin, and the consumption was at a very low level. The countries of Western Europe, which are at the forefront in terms of wealth, prefer a diet containing animal protein, and the consumption of plant substitutes is the prerogative of poorer countries [52] including Poland [53]. A diet in which protein of animal origin is replaced with protein of plant origin also goes in line with the trend of balanced consumption [27,54,55], which should additionally convince the consumer in favor of consumption of pulses. More than 30% of the respondents followed a diet that was dictated not only by health reasons but also by ethical or ecological beliefs, which is consistent with the conclusion of the results presented in the study by Honkanen et al. [56]. Wawryka et al. indicate, in the recommendations, an increase of the content of pulses in the diet due to their health-promoting properties and taste qualities [57]. The recommendations suggested that natural concerns, as well as motives related to health and weight control, were important for adopting and maintaining a potentially more balanced and healthy diet [20,58,59,60,61]. In the model proposed by the authors, “nutrition and health benefits of pulse products” was the highest-rated variable determining the motives of consumption. The results obtained from the research conducted by Rejman et al. [62] among the inhabitants of urban agglomerations confirmed that it is mostly women who make the decisions, in Polish society, about eating habits in households. 

Nutritional communication and consumer education on pulses is difficult [63]. The barriers of consumption reported by Polish consumers are similar to those observed among the residents of the USA, where, like other European respondents, they indicated a lack of knowledge about the use of pulses in dishes and their taste or consistency as not attractive [64]. Moreover, other studies conducted among the high-income countries indicate more practical concerns. Taste or aversion to pulses was a major obstacle among Canadian [65] and French consumers [66], as well as the lack of skills in preparing them or difficulties in cooking them.

The results suggest that for vegetable proteins to replace meat, some new knowledge and skills are needed in preparing and consuming pulse-based dishes. The results obtained in Scandinavia are similar to the results obtained in Poland [51,67]: To persuade consumers to change their eating habits, knowledge and skills are needed to prepare tasty meals, and various culinary programs run by recognized chefs would be helpful here. 

The duplication of purchasing channels [68,69] allowed us to determine which channel could be replaced with the main purchasing channel. During the ongoing pandemic the importance of substitute channels has increased, and the above-quoted results of the research on the pulse products market in Poland has shown possible substitute channels. It should be mentioned that the importance of the internet is increasing among purchasing channels. Online purchase of food products is declared by over 25% of Poles [70,71]. According to the research of the author, more than 15% of respondents purchase pulse products online, which accounts for more than 50% of all food purchased online. 

This research allowed us to determine the preferences of Polish people regarding products of pulse origin (level of processing), as well as to what extent the trends of the world cuisines influence decisions related to the consumption of those products. A wider range of products inspired by the cuisines of different cultures helps to introduce traditional pulse dishes, which could lead to an increase in the awareness of European consumers of their extremely varied use. According to the research conducted among Australian consumers, it was particularly Mexican, Indian, or Middle Eastern cuisine that had a positive effect on the perception of consumers on pulses [72]. Polish consumers are more encouraged to consume pulses by Middle Eastern and Mediterranean cuisine. This may be the result of the preferences of tourist destinations, where the consumer can usually become more familiar with the dishes of different cuisines of the world. In the updated Mediterranean dietary pyramid (MDP) the priority is given to the sources of plant protein, such as pulses, although the sources of animal protein that are low in saturated fat, such as fish, poultry, rabbit, and some lean meat, as well as eggs, are allowed in reasonable quantities. The daily intake of plant protein should be given priority, it ought to be at least one small portion per day. In the modified pyramid, it can be noticed that pulses are included in the daily consumption category. There are no reasons detrimental to the health to restrict pulses, but there are many environmental motives to increase their consumption. Pulses can replace animal protein-based foods in the diet, reducing the current bad impact of their production on the environment. The versatility of pulses increases their culinary value [44]. 

Referring to the research of Rybowska [73], the soy food products market can be evidence of the presented results, which concerned a wider range of assortment, that soybean as a pulse product attracts attention, and consumers buy them eagerly and more frequently; however, the knowledge of people about the health-promoting properties of soybean is not satisfactory. The demand for soy food products is also influenced by the easiness and speed of their preparation for consumption, as well as their popularity in the Middle Eastern cuisine, which is one of the cuisines influencing the consumption patterns of Poles. The barriers to purchasing soy food products, as indicated in the research, are the prices and still insufficient availability of them, limited only to selected points of sale, which may cause the duplication of sales channels. Consumer education is also required and it should be done through various social media feeds, as well as the information available in the institutions that spread the overall knowledge about the health benefits of food products.

### Limitations

The main limitation was the random selection of the sample, which on the one hand correctly reflected the populations according to Statistics Poland (Appendix A), but did not reflect the diet of the society. The diets “No specific diet”, “Limited animal products”, “Gluten free”, “A special diet related to medical conditions”, and “Other” were those most frequently indicated by consumers in the pilot study. The limitations may have also resulted from the way the tests were conducted. Online surveys are a very useful research tool because they can reach a wider audience. However, the computer system that is used in the research process cannot replace direct contact between the interviewer and the respondent. In future research, it is suggested to use the CATI method, where the interviewer contacts the respondent by phone.

## 5. Conclusions

The study examined the attitudes towards pulses as well as barriers and motives for their introduction or increasing their role in the daily nutrition of consumers. Despite the awareness of the health values of pulses, the image of those nutritionally and environmentally valuable products was influenced by the lack of habits of their preparation and the feeling of discomfort after eating them (e.g., intestinal movements). Most of the participants of the research, especially women, were positive about increasing the consumption of pulse products. It also turned out that women were more willing to replace meat with pulses.

The awareness that pulses are a source of protein seems insufficient for more respondents to believe that those products can replace meat in everyday cooking. This may be related to the cultural understanding of pulses in Poland. The well-known and recognizable format of meals in our part of Europe and other countries includes basic products such as potatoes, vegetables, and the protein component, which is usually meat. Promotional campaigns could also focus on teaching people how to make small and manageable changes in their daily food choices, which have proven to be the key to successful dietary changes. In the long term, this could also help people accept meat consumption reduction strategies and encourage them to reflect on the sustainability of their eating patterns.

## Figures and Tables

**Table 1:** Demographic description of the sample (*n* = 1067).

Variable	Category	Frequency	Percentage (%)
Gender	Male (M)	509	47.70
	Female (F)	558	52.30
Age	<25	312	29.24
	26–40	238	22.27
	41–55	299	28.02
	>56	218	20.47
Place of residence	Rural areas (rural communes)	420	39.40
	Cities of up to 30,000 inhabitants (urban–rural communes)	192	18.00
	Cities more than 30,000 inhabitants (cities)	455	42.60
Level of formal education	Primary	341	32.00
	Secondary	393	36.80
	University	333	31.20
Diet used	No specific diet (Profile A)	718	67.29
	Limited animal products (Profile B)	205	19.21
	Gluten free (Profile C)	102	9.56
	A special diet related to medical conditions (Profile D)	16	1.50
	Other (Profile E)	26	2.44

Source: Author’s own analysis based on study material.

**Table 2:** The frequency with which meals containing pulses are consumed.

Variable/Legume Product	Total Sample	Profile According to the Diet Used					*t*-Test	*p*-Value
	*n* = 1067	A	B	C	D	E		
Peas	2.69	2.15	3.90	3.58	3.96	3.87	20.17	<0.000 *
Beans	2.72	2.32	3.54	3.45	3.76	3.80	16.55	<0.000 *
Lentils	2.37	1.76	4.04	2.82	4.14	3.37	22.77	<0.000 *
Soybean	2.29	1.81	3.63	2.44	3.84	3.36	15.38	<0.000 *
Chickpea (*Cicer arietinum* L.)	1.91	1.41	3.05	2.57	3.05	3.36	17.47	<0.000 *
*Coronilla* L.	1.57	1.11	3.01	1.21	3.20	3.25	18.39	<0.000 *
Broad beans	1.47	1.10	2.24	1.81	3.24	3.12	10.41	<0.000 *
Average	2.14	1.66	3.34	2.55	3.60	3.45		

Note: * Level of significant difference at *p* < 0.050. Profile according to the diet used: A—No specific diet, B—Limited animal products, C—Gluten free, D—A special diet related to medical conditions, E—Other. Assessment: 1—I do not eat at all; 2—I eat it occasionally, less than once a week; 3—I eat it 1–2 times a week; 4—I eat it 3–4 times a week; 5—I eat 5 times or more. Source: Author’s own analysis based on study material.

**Table 3:** Motives for consuming meals containing pulse products among consumers.

Type of Motives	Wilks’ Lambda: 0.721 F = 4.327 *p* < 0.001 *			Sex	
	Wilks’ Lambda	F Value	*p*-Value	Female *p* = 0.373	Male *p* = 0.276
The products are healthy and nutritious	0.721	12.845	0.001 *	2.693	2.133
Pulse products are attractive in terms of their prices	0.791	8.809	0.021 *	2.416	2.085
They are meat substitutes and contain a lot of protein	0.702	7.281	0.045 *	2.876	2.091
They regulate blood sugar levels	0.711	5.410	0.089	1.404	1.501
They are part of my standard diet	0.687	3.357	0.214	1.168	1.345
Constants				12.729	11.531

Note: * Level of significant difference at *p* < 0.050. Source: Own analysis based on study material.

**Table 4:** Barriers to consuming meals containing pulse products among consumers.

Type of Barriers	Wilks’ Lambda: 0.732 F = 5.087 *p* < 0.001 *			Sex	
	Wilks’ Lambda	F Value	*p*-Value	Female *p* = 0.373	Male *p* = 0.276
Product taste and structure	0.771	7.845	0.001 *	1.693	1.319
Lack of knowledge and skills to prepare meals	0.751	5.809	0.006 *	2.186	2.005
Pulses contain a lot of carbohydrates	0.702	5.281	0.004 *	1.016	1.011
No such dietary habits	0.721	3.460	0.028 *	0.934	1.201
They cause bowel movements	0.718	3.357	0.044 *	0.908	0.745
Constants				8.292	8.161

Note: * Level of significant difference at *p* < 0.050. Source: Own analysis based on study material.

**Table 5:** Duplicating purchases between selected purchase channels for pulses products among consumers.

Purchase Channel	Total	A.	B.	C.	D.	E.	F.	G.	H.
A. On the farm	20.32		20.58	52.18	18.22	41.88	47.56	21.42	7.18
B. Producers’ stores	14.86	27.85		36.12	32.56	36.14	34.18	28.46	8.60
C. Markets, bazaars	35.20	34.46	18.12		32.40	34.22	41.15	29.16	6.82
D. Fairs, stalls	25.62	26.44	27.62	39.98		49.86	45.56	20.37	4.28
E. Festivals of local producers	32.75	32.16	25.48	48.27	48.46		38.32	22.45	5.38
F. Specialized stores “Healthy food—I Love Vege”	42.28	22.12	32.46	36.48	31.78	29.88		16.96	15.44
G. Large distribution networks	28.60	18.14	18.16	19.14	16.18	11.14	38.68		9.46
H. Internet	15.34	20.06	24.18	42.76	24.28	16.46	32.14	12.18	
Average duplication		25.89	23.80	39.28	29.13	31.36	39.66	21.57	8.16

Note: Total—The proportion of respondents reporting using a given purchase channel. Duplication can be averaged across purchase channels. The respondents could indicate at least 3 channels. Channel names from the columns: A.—On the farm, B.—Producers’ stores, C.—Markets, bazaars, D.—Fairs, stalls, E.—Festivals of local producers, F.—Specialized stores “Healthy food—I Love Vege”, G.—Large distribution networks, H.—Internet. Source: Own analysis based on study material.

**Table 6:** The form of pulse products purchased by consumers.

Variable	Female (%)	Male (%)	*t*-Test	*p*-Value
Fresh	20.61	11.00	1.625	0.389
Frozen	71.33	23.58	1.508	0.159
Dried	58.39	19.25	2.778	0.005 *
Canned	76.16	54.62	2.759	0.003 *
In prepared dishes	65.41	74.26	3.895	<0.000 *

Note: * Level of significant difference at *p* < 0.050. The respondents indicated at least 3 forms of the most frequently purchased products. The percentage of male and female buying legumes was calculated on the basis of female *n*—558, male *n*—509.

**Table 7:** The relationship between the culinary trend and the tendency to buy and consume pulse products/dishes.

Factors	Estimate (β)	Standard Error	*p*-Value
Mexican cuisine (A)	0.107	0.175	<0.001
Indian cuisine (B)	0.086	0.038	0.037
Middle Eastern cuisine (C)	0.120	0.181	0.012
Mediterranean cuisine (D)	0.113	0.102	<0.001
Other (E)	0.061	0.049	0.036
F- statistic of the model	F(1.864) = 0.562		
Constant	2.587		
Se	1.315		
Coefficient of determination (R^2^)	32.2%		

Note: Level of significant difference at *p* < 0.050. Source: Author’s own analysis based on study material.

## Data Availability

The data presented in this study are available on request from the corresponding author.

## Appendix A

**Table A1:** Comparison of the Sample with the General Population (%).

Variable	Category	General Population	Study Sample
Gender	Male (M)	47.90	47.70
	Female (F)	52.10	52.30
Age	<25	30.30	29.24
	26–40	20.80	22.27
	41–55	26.80	28.02
	>56	22.10	20.47
Place of residence	Rural areas (rural communes)	40.60	39.40
	Cities of up to 30,000 inhabitants (urban–rural communes)	20.10	18.00
	Cities more than 30,000 inhabitants (cities)	39.30	42.60
Level of formal education	Primary	34.30	32.00
	Secondary	35.90	36.80
	University	29.80	31.20
